# Bilateral Thalamic Stroke Following Cardiac Arrest Due to Massive Saddle Pulmonary Embolism

**DOI:** 10.7759/cureus.101964

**Published:** 2026-01-21

**Authors:** Abay Gobezie, Kristin N Slater, Haregua Zenebe, Mahburbur Sumon

**Affiliations:** 1 Internal Medicine, Howard University Hospital, Washington, D.C., USA; 2 Pulmonary and Critical Care, Howard University Hospital, Washington, D.C., USA

**Keywords:** cardiac arrest, pe, pulmonary embolism, stroke, thalamic stroke

## Abstract

Bilateral thalamic infarctions are rare, but can be seen in patients experiencing occlusion or hypoperfusion. Saddle emboli can be very dangerous, leading to cardiac arrest. The occurrence of both in a single patient is an incredibly rare and dangerous entity, infrequently found in the literature. We present a case of a bilateral thalamic stroke in a 55-year-old male who experienced cardiac arrest secondary to a massive saddle pulmonary embolism. This case highlights the rare occurrence of bilateral thalamic infarction in the context of global cerebral hypoperfusion following cardiopulmonary compromise.

## Introduction

Bilateral thalamic infarction is a rare entity, often resulting from specific vascular insults, such as occlusion of the artery of Percheron or global hypoperfusion [1-10]. The thalamus, being a critical relay center for sensory and motor pathways [1], can present with diverse clinical manifestations when affected. The presentation can range, including, but not limited to, bilateral vertical gaze palsy [1-7], cognitive impairments [1-7,9-10], sleep disturbances, specifically hypersomnolence [1-2], and motor defects [1,4-5,9-10]. Infarctions of this nature have a poor prognosis and can be difficult to diagnose [1-3,6-7,9-10].

Saddle emboli are a very dangerous entity that can lead to cardiac arrest [11-13]. The gravity of the diagnosis cannot be understated, as the presenting symptom of a saddle embolus can be sudden death [11-13]. Located at the bifurcation of the pulmonary artery [11,12], these types of emboli can cause significant right heart strain [11,13]. While uncommon, patients with a saddle embolism have a high mortality rate [11,12].

To our knowledge, there are no reported cases documenting bilateral thalamic infarction in the setting of a massive saddle embolism. This report discusses the unique presentation and challenges associated with bilateral thalamic infarction in the context of global cerebral hypoperfusion following cardiopulmonary compromise.

## Case presentation

A 55-year-old African American male with a history of hypertension, hyperlipidemia, and poorly controlled diabetes mellitus (HbA1c > 15.5 within the last year, reference range <5.7%) was admitted to the medical intensive care unit (MICU) following an out-of-hospital cardiac arrest. The patient’s family had witnessed the patient collapse at home after he had indicated that he felt “funny” earlier that day. The family called EMS, which arrived within 10 minutes. In total, about 15 minutes had elapsed between the patient collapsing and EMS starting cardiopulmonary resuscitation (CPR) after arriving to find the patient lying supine, in cardiac arrest on the ground in the doorway of the home. Manual chest compressions were started, and the patient was ventilated with bag-valve-mask ventilation with supplemental oxygen. The first monitored cardiac rhythm was pulseless electrical activity (PEA). Blood glucose reading in the field showed “high.” An advanced life support (ALS) transport unit was requested, and an inline capnography was connected. Between cycles of CPR, an automatic compression device was placed, and right humeral head intraosseous (IO) cannulation was achieved after access in the left humeral head failed. The first 1 mg of epinephrine was given upon achieving IO access, and every five minutes moving forward while the patient was in cardiac arrest. Fluids were started, and on the third pulse check, return of spontaneous circulation (ROSC) was achieved with an initial blood pressure reading of 190/90 mmHg. However, the following blood pressure reading dropped to 82/55 mmHg, and an epinephrine 10 mcg push was given to support the patient’s blood pressure. The patient was transported in the ambulance, but en route to the hospital, he went into a second round of cardiac arrest with a PEA rhythm. Chest compressions were resumed by an automatic compression device, and an additional 1 mg of epinephrine was given. ROSC was achieved again; however, the patient became hypotensive, and an additional 10 mcg push of epinephrine was given for blood pressure support. He arrived at the Emergency Department (ED) in ROSC with a pulse of approximately 100. In the ED, he was intubated. Initial labs revealed significantly elevated lactic acid, consistent with hypoperfusion; elevated beta-hydroxybutyrate and glucose, consistent with diabetic ketoacidosis; a high-sensitivity troponin level of 31 pg/mL (which uptrended to a peak of 1223 pg/mL), likely due to demand ischemia; and a low hemoglobin level (Table 1). His initial arterial blood gas (ABG) showed profound acidosis with a pH of 6.936 NM, Bicarb of 18.59 NM, PCO2 of 86.5 NM, and PO2 of 220.8 NM. On arrival, white blood cell (WBC) counts and absolute neutrophil counts (ANCs) were within normal limits. He was transferred to the MICU for further management.

**Table 1:** Initial pertinent labs. The patient's relevant labs on presentation with reference ranges.

Lab	Initial Lab Value	Reference Range
Beta-hydroxybutyrate	47.6 mg/dL	0-4.5 mg/dL
Anion gap	23 mEq/L	7-16 mEq/L
Glucose	575 mg/dL	70-100 mg/dL
High sensitivity troponin	31 pg/mL	0-20 Ng/L
Lactic acid	9.5 mm/L	0.5-2.2 mm/L
Hemoglobin	9.8 g/dL	14.6-17.8 g/dL
White blood cell count	9.82 K/mcL	3.2-10.6 K/mcL
Absolute neutrophil count	4.07 K/mcL	1.3-7.1 K/mcL

**Figure 1:**
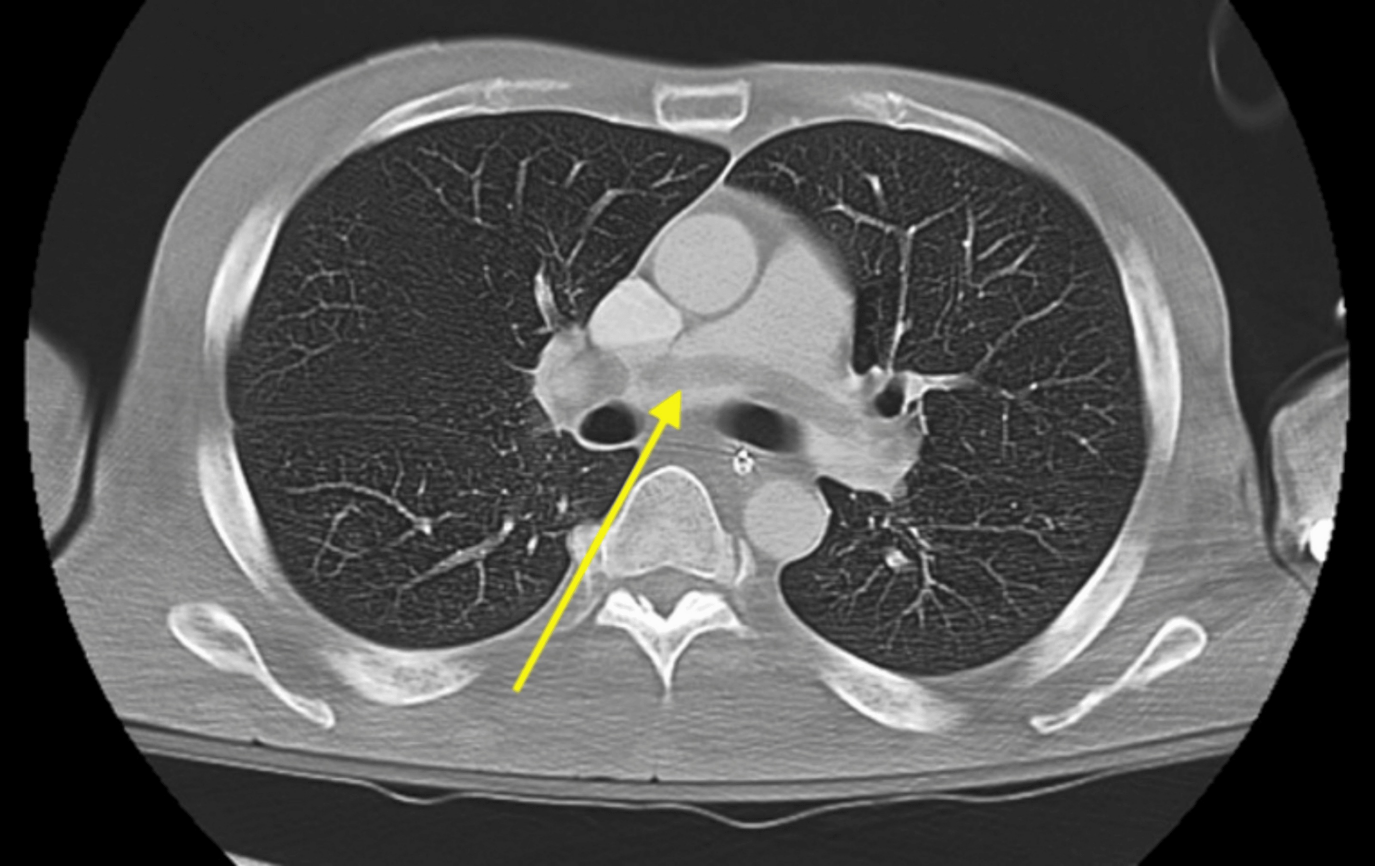
Massive saddle embolism seen on computed tomography pulmonary angiography.

He became hypotensive, requiring pressors (norepinephrine and vasopressin), and his diabetic ketoacidosis required an insulin drip. Imaging was obtained, and the initial X-ray of the chest findings were consistent with persistent right lower lobe airspace disease and small pleural effusion. The patient was tachycardic with a D-dimer level of >20 µg/mL on presentation. Computed tomography pulmonary angiography (CTPA) of the chest was completed, which showed a massive saddle pulmonary embolism, and an alteplase 50 mg drip was started (Figure 1). Cardiothoracic surgery was consulted, who agreed with the plan to proceed with tPA 50 mg IV over two hours, followed by either heparin at fixed doses of 500 or 100 U/hour or a target antiXa range of 0.2-0.5 U/mL or a goal PTT of 40-60 seconds per ELSO guidelines. He was started on a heparin drip, following the above parameters. PTT within the range of 40-60 seconds was maintained. Cardiology was consulted, who completed an echocardiogram, showing right heart strain.

He continued to require pressors, his WBC count began to rise, peaking at 16.93 K/mcL, and his ANC increased, peaking at 13.14 K/mcL. Peripheral blood cultures resulted in *Staphylococcus lugdunensis*, and intravenous (IV) cefazolin 2 g every eight hours and IV metronidazole 500 mg every eight hours were initiated. His WBC and ANC normalized with antibiotic treatment, and his pressor requirements lessened, improving his septic shock.

**Figure 2:**
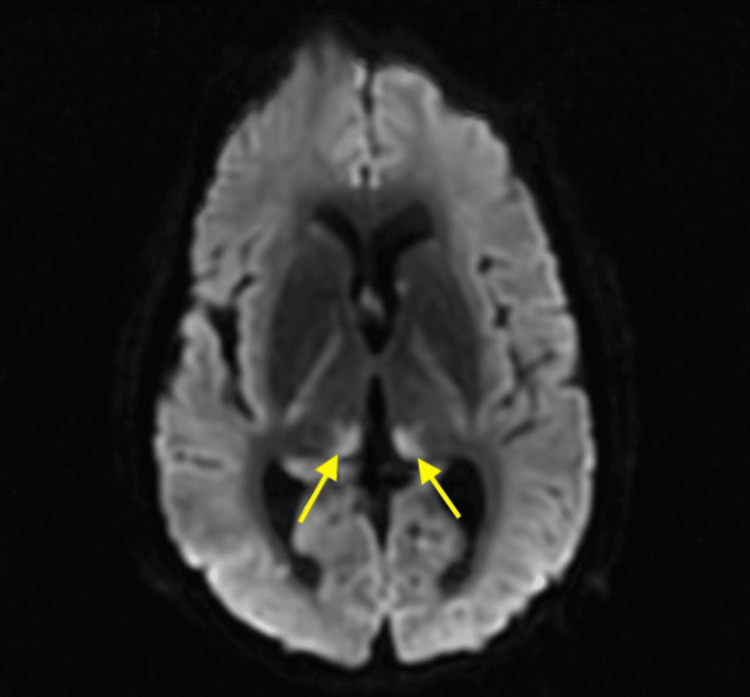
MRI diffusion-weighted imaging (DWI) of the brain revealed acute bilateral paramedian thalamic infarcts, indicated by the yellow arrows.

**Figure 3:**
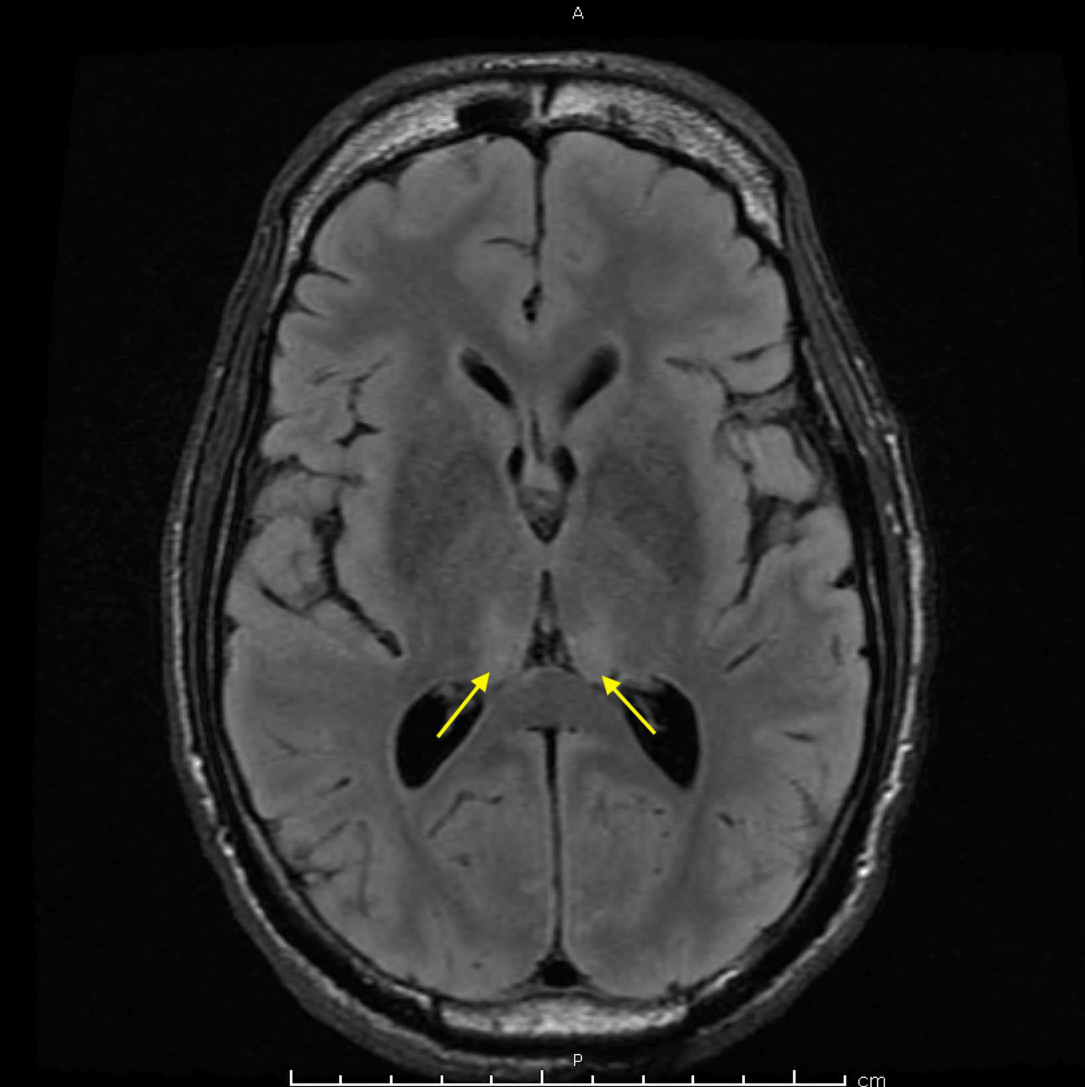
MRI fluid-attenuated inversion recovery (FLAIR) of the brain revealed acute bilateral paramedian thalamic infarcts indicated by the yellow arrows.

Neurology was consulted with an initial neurological assessment that showed absent corneal and gag reflexes, and absent doll eye movement. The pupils were small, measuring about 1 mm, equal, bilateral, and reactive. The clinical examination indicated significant dysfunction of the brainstem function apart from small pupillary reflexes. EEG was completed, which showed generalized suppression. The findings were suggestive of profound generalized cerebral dysfunction, which can be seen with post-anoxic brain injury, worse on the left. No seizures were recorded. Computed tomography angiography (CTA) of the head and neck was attained, showing radiographic evidence of cerebral edema and heterogeneous brain parenchyma, possibly indicating infarction. Magnetic resonance imaging (MRI) of the brain revealed acute bilateral paramedian thalamic infarcts associated with possible occlusion of the artery of Percheron and increased diffusion restriction in the bilateral medial thalami, consistent with acute infarction, and mild restriction in the bilateral hippocampal regions, suggestive of hypoxic-ischemic injury. Scattered foci of restriction in the frontal lobes were noted but attributed to T2 shine-through artifact (Figures 2-3).

Despite therapeutic efforts, the patient showed minimal neurological improvement. Palliative care was involved in goals-of-care (GOC) discussions. By MICU day 10, the patient’s code status was changed to do not resuscitate (DNR), with ongoing family deliberations regarding tracheostomy and percutaneous endoscopic gastrostomy (PEG) tube placement. At that time, the patient remained comatose with significantly impaired brainstem function. His ABG trends, however, showed consistent improvement in oxygenation (PaO2/FiO2 ratio ~220). Despite this, his prognosis remained very poor. Ultimately, on MICU day 14, the patient remained in critical condition, and during a GOC discussion with the family, they ultimately chose to further change his code status to do not intubate (DNI) with compassionate extubation. The patient was extubated and expired within an hour of compassionate extubation with family by his side.

## Discussion

Bilateral thalamic strokes are uncommon and can result from global hypoperfusion or embolic events involving the artery of Percheron [1-10]. In this case, prolonged cardiac arrest likely caused diffuse hypoxic-ischemic injury, with selective vulnerability of the thalamus due to its high metabolic demand. The pathophysiology of global hypoperfusion can lead to selective vulnerability of deep brain structures, including the bilateral thalami, which is a distinct entity from embolic or thrombotic events (such as an embolism of the artery of Percheron) [1-10]. Diagnosis of bilateral thalamic strokes is best made through MRI with diffusion-weighted imaging. In our imaging, the differential of a possible occlusion of the artery of Percheron was noted. However, in the context of this case, the bilateral thalamic infarctions were unlikely to be embolic and more likely to be due to global ischemia following cardiopulmonary compromise due to his massive saddle embolism and cardiac arrests. The management of the global hypoperfusion caused by a massive saddle embolism is prompt revascularization and neuroprotection, which are essential, but outcomes depend heavily on the duration and severity of hypoxia.

The occurrence of these two very dangerous and likely related diagnoses is rare. This case shows the possibility of their acute and related coexistence. His risk factors include his history of poorly controlled diabetes, hypertension, and hyperlipidemia. Table 2 outlines a comparison of patient demographics, past medical history, and outcomes of the cases of bilateral thalamic infarcts sourced in this article (Table 2). When compared to the cases cited in this report, our patient’s age (55 years of age) was on the younger end of the range reported for bilateral thalamic infarcts (range 48-83 years of age, mean age: 63.85 years of age). The sourced articles did not commonly list race as a part of their case description; however, we have included it, as it is known that race and social vulnerability have both been shown to play a significant role in diabetes control [14]. In our case, uncontrolled diabetes was likely a significant risk factor for our patient. Common risk factors in our sources and in our patients included hypertension, diabetes, and hyperlipidemia. In the 13 cases in our citations, there was a male predominance of 9:4. Our patient’s outcome was worse than the reported outcomes, which is expected, due to his two episodes of cardiac arrest prior to arriving at the hospital, the presence and severity of his saddle pulmonary embolus, combined with his bilateral thalamic infarct, likely secondary to hypoperfusion. 

**Table 2:** Bilateral thalamic stroke demographics, medical history, and reported outcomes. Table summary of the cases sourced in this article, organized by source, demographic information, medical history, presentation, and reported outcomes.

Source	Age	Sex	Medical History	Presentation	Outcome
Bhattarai et al., 2023 [1]	60 years old	Female	Hypertension	Loss of consciousness	Neurological deficits including left sided hemiplegia and severe cognitive impairment [1].
Yamagushi and Yakushiji, 2021 [2]	81 years old	Male	Unknown	Impaired consciousness	Persistent somnolence with variable Glasgow Coma Scale scores of 8-13, and disorientation to time and place. Transferred to sanatorium [2].
Lahnine et al., 2024 [3]	64 years old	Male	None	Sudden coma	Confused but awake and following minimal commands in first 48 hours. Continued to show improvement, passed a speech and swallow test. Ultimately, discharged to subacute rehab with neurological follow-ups [3].
Achhami et al., 2023 [4]	48 years old	Male	Diabetes	Diplopia, bilateral drooping of eyelids and loss of consciousness while working, fever on presentation of 101°F	Home care, still experiences difficulty in opening eyes spontaneously, now showing positive signs of responsiveness by communicating verbally and demonstrating spontaneous movement in all their limbs [4].
Shams et al., 2021 [5]	58 years old	Male	Hypertension	New-onset slurring of speech and involuntary spilling of water during an attempt to drink	Back to baseline, discharged home with outpatient follow up [5].
Chen et al., 2021 [6]	57 years old	Male	Hypertension	Dizziness and blurred vision when working, collapse followed by unconsciousness for 2 days	Discharged, with persistent cognitive dysfunction, memory decline, mental decline, vertical fixation paralysis, no fluency in speech and unstable emotions [6].
Chen et al., 2021 [6]	57 years old	Male	None	Found unconscious by family able to answer questions inaccurately on presentation, unable to move limbs, slurred speech	MMSE of 27 (secondary school level) at discharge, persistently poor memory and calculation ability at 1 year follow up [6].
Alaithan et al., 2023 [7]	58 years old	Female	Hypertension, hyperlipidemia	Sudden onset confusion, inability to speak, and right-sided weakness, disorentation	Discharged home after rehabilitation with mild hemiparesis, Rankin Scale (mRS) of 2, slight disability but able to live without assistance, but unable to continue all previous activities [7].
Qureshi et al., 2021 [8]	77 years old	Male	Gout, hypertension	Episode of right facial droop and speech slurring. Capable of localizing to pain but had a decreased level of consciousness on presentation	Unknown [8]
Donohoe et al., 2022 [9]	56 years old	Female	Nonischemic cardiomyopathy, hypertension, COPD, hyperlipidemia, left vevntricular thrombus on rivaroxaban, depression, anxiety, and polysubstance use disorder	Acute encephalopathy of unknown cause, desaturated to 84% oxygen with an episode of apnea, and hypertensive to 152/100 mmHg	Mental status failed to improve, cognitive evaluation 0/5. Developed dysphagia requiring PEG tube, guardianship was instituted due to incapacity. Ultimately discharged to skilled nursing facility [9].
Li et al., 2023 [10]	72 years old	Female	Hypertension, diabetes mellitus, coronary heart disease, unstable angina pectoris, and atrial fibrillation without anticoagulation after percutaneous coronary intervention (PCI)	Sudden onset of unclear speech, impaired consciousness, and right-sided weakness	Unknown [10]
Li et al., 2023 [10]	83 years old	Male	Kimura's disease, hyperlipidemia, hypertension, and diabetes mellitus	Paroxysmal vertigo, nausea, and tinnitus affecting both ears without hearing impairment	Unknown [10]
Li et al., 2023 [10]	59 years old	Male	Impaired glucose tolerance	Sudden slurred, slow speech and somnolence	Unknown [10]

This case acts as a reminder to be vigilant in screening for saddle emboli and underscores the need to consider all of the ramifications that can come from such a dangerous diagnosis. MRI of the brain to search for global hypoperfusion is critical; however, outcomes will vary depending on the amount of time the patient endured ischemia. Ultimately, compassionate extubation was chosen by the family for our patient, as his prognosis was incredibly poor given multiple risk factors, including approximately 15 minutes of unresponsiveness before CPR was initiated and the two rounds of CPR predating his arrival to the hospital. Each of his diagnoses (saddle embolism and bilateral thalamic infarction in the setting of global cerebral hypoperfusion) made his prognosis very poor [1-13]. 

## Conclusions

This case underscores the importance of recognizing rare neurovascular complications, such as bilateral thalamic strokes, in the context of cardiac arrest and global hypoperfusion. Early neuroimaging and a multidisciplinary approach are critical for optimal management, although the prognosis remains guarded in such cases. In cases like this, with poor prognostic outcomes, having early and consistent GOC conversations with family to aid in processing, grieving, and expectations can be valuable. It also highlights the importance of preventative care in mitigating and controlling common diseases that serve as risk factors for vascular complications such as diabetes, hypertension, and hyperlipidemia.
